# Effects of Selenylation Modification on Immune-Enhancing Activity of Garlic Polysaccharide

**DOI:** 10.1371/journal.pone.0086377

**Published:** 2014-01-30

**Authors:** Shulei Qiu, Jin Chen, Tao Qin, Yuanliang Hu, Deyun Wang, Qiang Fan, Cunshuai Zhang, Xingying Chen, Xiaolan Chen, Cui Liu, Zhenzhen Gao, Xiuping Li

**Affiliations:** 1 Institute of Traditional Chinese Veterinary Medicine, College of Veterinary Medicine, Nanjing Agricultural University, Nanjing, China; 2 National Research Center of Veterinary Biological Engineering and Technology, Jiangsu Academy of Agricultural Sciences, Nanjing, China; 3 China Institute of Veterinary Drug Control, Beijing, China; University of Florida College of Medicine, United States of America

## Abstract

The garlic polysaccharide was modified by HNO_3_-Na_2_SeO_3_ method according to orthogonal design L_9_(3^4^) to obtain nine selenizing garlic polysaccharides, sGPS_1_-sGPS_9_. Their effects on chicken peripheral lymphocytes proliferation in vitro were compared by MTT assay. The results showed that sGPSs could significantly promote lymphocytes proliferation, sGPS_3_, sGPS_5_ and sGPS_6_ presented stronger efficacy. In vivo experiment, 14-day-old chickens were injected respectively with sGPS_3_, sGPS_5_ and sGPS_6_ when they were vaccinated with ND vaccine taking unmodified GPS as control. The results showed that three sGPSs could significantly promote lymphocyte proliferation, enhance serum antibody titer, IFN-γ and IL-2 contents. These results indicated that selenylation modification could significantly enhance the immune-enhancing activity of GPS, sGPS_6_ possessed the best efficacy and could be as a candidate drug of immunoenhancer. Its optimal modification conditions were 400 mg of sodium selenite for 500 mg of GPS, reaction temperature of 70°C and reaction time of 6 h.

## Introduction

Garlic is an eatable plant and also used as medicinal herb in many countries of the world [1], [2]. It contains the water of 62%–68%, carbohydrate of 26%–30%, protein of 1.5%–2.1%, amino acids of 1%–1.5% and so on [3]. Garlic polysaccharide (GPS) is one of the major medicinal components of garlic and possess a variety of bioactivities, such as enhancing immunity, anti-bacterium, anti-virus, anti-oxidation, hepatoprotective, hypolipidemic, hypoglycemic and so on [4]–[6].

Selenium is an essential trace element for organisms [7]. In most districts of the world, people and animals need supplementing selenium. The selenium source includes organic and inorganic selenium. When inorganic selenium is applied, its dose is difficultly controlled and it has the disadvantage of cumulative toxicity and mutagenic effects. While organic selenium has lower toxicity and fewer side effects, it can not only play a better role of selenium, but also more significantly stimulate immune responses in comparison with inorganic selenium [8]. Because the organic selenium source is relatively rare, the combination of selenium with polysaccharide into organic selenium compounds, selenizing polysaccharide, is not only an effective measure to develop new selenium source, but also make the physiological and pharmacological function of selenium and polysaccharide be optimized. Many researches confirmed that selenizing polysaccharide possessed duple and higher biological activity of polysaccharide and selenium, and was easily absorbed and utilized by the organism [7]. Therefore, it has very important significance to research the biosynthesis and pharmacological activity of selenizing polysaccharide [9].

The selenylation modification method of polysaccharide includes nitric acid–sodium selenite (HNO_3_-Na_2_SeO_3_) method [10], Selenium oxidate–pyridine method [11]–[13] and so on. Among these methods, HNO_3_-Na_2_SeO_3_ method is commonly used since the reaction conditions are simpler, production is faster and selenium content of modifier is higher [14].

In this study, GPS was modified by HNO_3_-Na_2_SeO_3_ method under nine kinds of modification conditions selecting three factors, usage amount of sodium selenite, the reaction temperature and reaction time [15], at three levels according to L_9_(3^4^) orthogonal design. Nine selenizing GPSs (sGPSs), sGPS_1_-sGPS_9_ were obtained. Firstly, the effects of nine sGPSs on chicken peripheral lymphocytes proliferation in vitro were compared by MTT assay. Three sGPSs with better effect were picked out then injected for the chickens vaccinated with ND vaccine. The lymphocyte proliferation, serum antibody titer, IFN-γ and IL-2 contents of the chickens were determined. The aim of this study is to explore the probability of selenylation modification to improve the immune-enhancing activity of GPS, selected out the best sGPS and modification condition, and offer theoretical evidence for the development of new-type immunopotentiator.

## Materials and Methods

### Reagents and vaccine

Sodium selenite bought from Shanghai Lingfeng Chemical Reagent Ltd. was dissolved into 0.05 g·mL^−1^ with ultrapure water. Standard selenium stored solution at 100 µg·mL^−1^ supplied by National standard substance research center was accurately diluted into 1 µg·mL^−1^ of standard selenium solution. Nitric acid (HNO_3_) was the product of Shanghai Lingfeng Chemical Reagent Ltd.

RPMI-1640 (GIBCO) supplemented with 100 IU·mL^−1^ benzylpenicillin, 100 IU·mL^−1^ streptomycin and 10% fetal bovine serum was used for washing and re-suspending cells, diluting mitogen and culturing the cells. Hanks' solution, pH was adjusted to 7.4 with 5.6% sodium bicarbonate solution, supplemented with benzylpenicillin 100 IU·mL^−1^ and streptomycin 100 IU·mL^−1^, was used for diluting blood. The 3-(4,5-dimethylthiazol-2-yl)-2,5-diphenyltetrazolium bromide (MTT, American Co.) was dissolved into 5 mg mL^−1^ with calcium and magnesium-free (CMF) phosphate-buffered saline (PBS, pH 7.4). Sodium heparin was dissolved into 5 mg mL^−1^ with PBS. These reagents were filtered through a 0.22 µm syringe filter. Sodium heparin solution were stored at −20°C, the others were at 4°C and MTT solution was in dark bottle.

Dimethylsulfoxide (DMSO, No. 12080211263), Nitric acid (HNO_3_) and Sodium selenite was the product of Shanghai Lingfeng Chemical Reagent Ltd.. Lymphocytes Separation Medium (No.20121201) was manufactured by Shanghai Hengxin Chemicals Ltd.

Newcastle Disease vaccine (La Sota strain, No. 119082) was purchased from Institute of Veterinary Medicine, Jiangsu Academy of Agricultural Science.

Chickens Interferon-γ (IFN-γ) ELISA kits and Chickens Interleukin-2 (IL-2) ELISA kits were the products of Shanghai Langdun Biotechnology Inc.

### Preparation of sGPS

#### 1. Extraction and purification of GPS

Garlic was bought from a farm product market of Nanjing City, place of production was Shandong province of China. GPS was extracted by water decoction and ethanol precipitation, purified by trichloroacetic acid method to eliminate protein, through column chromatography of DEAE Cellulose-52 (1.6 cm×50 cm) and eluted with distilled water. The flow rate was maintained at 0.8 mL·min^−1^, the eluent was collected with automatic fraction collector, 8 mL per tube [16]. The carbohydrate was examined by phenol-sulfuric acid assay and elution curve was drawn (to get a peak). The eluents contained polysaccharides were mixed, concentrated under reduced pressure and freeze-dried to obtain the purified GPS. Its carbohydrate content was 94.5% determined by the phenol-sulfuric acid method [17], [18].

#### 2. Selenylation modification of GPS

Three factors respectively at three levels, the reaction temperature at 50, 70 and 90°C (A), the usage amount of sodium selenite at 200, 300 and 400 mg for 500 mg of GPS (B), and the reaction time for 6, 8 and 10 h (C), were selected [15]. Nine modification conditions were designed according to L_9_ (3^4^) orthogonal test (Table 1). After the reaction was finished, the mixture was cooled to room temperature, adjusted pH to 5–6 with saturated sodium carbonate solution, dialyzed in dialysis sack with 1 kDa ultrafiltration membrane against tap water and till no free sodium selenium detected by ascorbic acid method [15], and the polysaccharide solution was concentrated and freeze-dried. Nine selenizing GPSs respectively named sGPS_1_–sGPS_9_ were obtained. Their selenium contents were determined by atomic fluorescence spectrometry [19] with atomic fluorescence spectrometer (Model AFS-930, Beijing Jitian instrument Co., Ltd.), and the carbohydrate contents were determined by the phenol-sulfuric acid method.

**Table 1:** Modification conditions, yields and contents of selenium and carbohydrate of every sGPS.

sGPS	A Temperature (°C)	B Na_2_SeO_3_(mg)	C Time(h)	Rate of Yeild (%)	Se content (mg·g^−1^)	Carbohydrate content (%)
sGPS_1_	50	200	6	36.90	6.94	46.4
sGPS_2_	50	300	8	37.64	8.37	45.7
sGPS_3_	50	400	10	27.65	12.49	45.2
sGPS_4_	70	200	8	32.10	9.43	49.3
sGPS_5_	70	300	10	30.25	10.45	56.9
sGPS_6_	70	400	6	34.43	11.36	52.6
sGPS_7_	90	200	10	18.10	6.21	21.2
sGPS_8_	90	300	6	24.32	7.99	34.4
sGPS_9_	90	400	8	19.30	8.76	24.7

For the test in vitro, nine sGPSs were dissolved into 0.2 mg·mL^−1^ with distilled water. For experiment in vivo, sGPS_3_, sGPS_5_, sGPS_6_ and GPS were diluted into 1 mg·mL^−1^ with distilled water. The diluted preparations were sterilized by pasteurization and detected for endotoxin by pyrogen tests. When the endotoxin amount was up to the standard of Chinese Veterinary Pharmacopoeia (less than 0.5 EU mL^−1^) [20], they were stored at 4°C for the test [21].

### Experimental animals

One-day-old White Roman chicken (male), purchased from Tangquan Poultry Farm, were housed in wire cages (40 cm×60 cm×100 cm) in air-conditioned rooms at 37°C and lighted for 24 h at the beginning of pretrial period. The temperature was gradually declined to the room temperature and the light time to 12 h per day, which was kept constant in the following days. Chickens were fed with the commercial starter diet provided by the feed factory of Jiangsu Academy of Agricultural Science. The later experimental procedures were performed in strict accordance with internationally accepted principles and Chinese legislation on the use and care of laboratory animals, and the experimental use of animals and procedures followed were approved by the Nanjing Agricultural University Animal Care Committee. All efforts were made to minimize suffering.

### In vitro test

The effects of nine sGPSs on chicken peripheral lymphocytes proliferation in vitro were determined by MTT assay [22]. sGPS_1_–sGPS_9_ were diluted with RPMI-1640 in twofold serially from 100 µg·mL^−1^ to 0.097 µg·mL^−1^, GPS, from 1000 µg·mL^−1^ to 0.97 µg·mL^−1^, total 11 concentrations. Blood samples were collected from sixty-day-old non-vaccinated chickens and transferred immediately into aseptic capped tubes with sodium heparin, then diluted with equal volume of Hanks' solution and carefully layered on the surface of lymphocytes separation medium. After 20 min of centrifuged at 2000 rpm, a white cloud-like lymphocytes' band was collected and washed twice with RPMI-1640 media without fetal bovine serum. The resulting pellet was re-suspended to 2.5×10^6^ mL^−1^ with RPMI-1640 media, and inoculated into 96-well culture plates, 100 µL per well. Then, in polysaccharide groups the ten polysaccharides at series of concentrations were added, 100 µL per well, four wells each concentration, in cell control group (CC), RPMI-1640 media of 100 µL. The plates were incubated at 38.5°C in a humid atmosphere of 5% CO_2_. After a incubation for 44 h, 20 µL of MTT (5 µg·mL^−1^) was added into each well, and continued to incubate for 4 h. The supernatant was removed carefully and 100 µL of DMSO were added into each well to dissolve the formazan crystals. The plates were shaken for 5 min to dissolve the crystals completely. The absorbance of cells in each well was measured by microliter enzyme-linked immunosorbent assay reader (Model DG-3022, East China Vacuum Tube Manufacturer) at a wave length of 570 nm (*A*_570_ value) [22], [23]. When the *A*_570_ values of polysaccharide group were not significantly lower than that of the cells control group, it indicated that the polysaccharides had no cytotoxicity, the corresponding concentrations were considered as its maximal safe concentration for lymphocytes. The experiment showed that the maximal safe concentrations of all sGPSs and GPS were within 500–3.125 µg·mL^−1^. Their safe concentrations were supposed as 3.125 µg·mL^−1^ in order to make the comparison at the same level.

sGPS_1_–sGPS_9_ and GPS were diluted with RPMI-1640 twofold serially from 3.125 µg·mL^−1^ to 0.097 µg·mL^−1^ total 5 concentrations. The preparation of lymphocytes as above, the resulting pellet was re-suspended into 2.5×10^6^ mL^−1^ with RPMI-1640 media, and respectively inoculated into 96-well culture plates, 100 µL per well. Then, in polysaccharide groups the ten polysaccharides at series of concentrations were added, 100 µL per well, four wells each concentration. At the same time, cell control group (CC, only adding RPMI-1640 media) was designed. The cellular *A*_570_ values were determined by above-mentioned method as the index of lymphocytes proliferation. Meanwhile the cellular proliferation rate was calculated to compare the strength of lymphocytes proliferation according to the equation (Yu, et al., 2005; Lu, 2008): Lymphocytes proliferation rate (%) = (*Ā*_polysaccharide group_–*Ā*_control group_)/*Ā*_control group_)×100% (*Ā* was a average value of five concentration groups of polysaccharide or four wells of control group).

### In vivo test

#### 1. Experimental design

One hundred and eighty 14-day-old chickens (average maternal ND-HI antibody titer was 2.8 log2) were randomly assigned into 6 groups. The chickens except for blank control (BC) group were vaccinated with ND vaccine, repeated vaccination at 28 days old. At the same time of the first vaccination, the chickens in four polysaccharide groups were intramuscularly injected respectively with 0.5 mL of sGPS_3_, sGPS_5_, sGPS_6_ and GPS, in vaccination control (VC) and BC group, with equal volume of physiological saline, once a day for three successive days.

#### 2. Peripheral lymphocytes proliferation assay

On days 7 (D_7_), 14 (D_14_), 21 (D_21_) and 28 (D_28_) after the first vaccination, the blood samples of four chickens randomly from each group were collected for the determination of peripheral lymphocytes proliferation by MTT assay (same to 2.3). The cellular *A*_570_ values were determined as the index of lymphocytes proliferation. Meanwhile the average lymphocytes proliferation rates were calculated to compare the strength of lymphocytes proliferation according to the equation (Yu, et al., 2005; Lu, 2008): Average lymphocytes proliferation rate (%) = (*Ā*_polysaccharide group_–*Ā*_BC group_)/*Ā*_BC group_)×100% (*Ā* was an average value of the four time points).

#### 3. Serum HI antibody assay

On days 7 (D_7_), 14 (D_14_), 21 (D_21_) and 28 (D_28_) after the first vaccination, the blood samples of six chickens randomly from each group were collected for examination of serum hemagglutination inhibition (HI) antibody titer by micro-method [24].

#### 4. Serum IFN-γ and IL-2 contents

On days 14 (D_14_), 21 (D_21_) and 28 (D_28_) after the first vaccination, the blood samples of six chickens randomly from each group were collected for determination of serum contents of IFN-γ and IL-2 by Enzyme-linked Immunosorbent Assay (ELISA).

### Statistical analysis

Data were expressed as means ± SEM and the Duncan's multiple range tests was used to analyze the difference among polysaccharides and control groups. Differences between means were considered significant at *P*<0.05.

## Results

### The yields and contents of selenium and carbohydrate in each sGPS

The modification conditions, yields and contents of selenium and carbohydrate of every sGPSs are listed in Table 1. The yield of sGPS_2_ was the highest, up to 37.64%, and next were sGPS_1_, sGPS_6_ and sGPS_4_. The selenium content of sGPS_3_ was the highest (12.49 mg·g^−1^), and next were sGPS_6_, sGPS_5_ and sGPS_4_. The carbohydrate content of sGPS_5_ was the highest (56.9%), and next were sGPS_6_, sGPS_4_ and sGPS_1_.

### The change of lymphocytes proliferation in vitro test

The cellular *A*_570_ values of every group are listed in Table 2. The *A*_570_ values in sGPS_1_ at 3.125–0.391 µg·mL^−1^, sGPS_2_–sGPS_6_, sGPS_8_–sGPS_9_ and GPS at all concentrations, sGPS_7_ at 1.563–0.195 µg·mL^−1^ groups were significantly larger than those of corresponding cell control group respectively (*P*<0.05).

**Table 2:** The cellular *A*_570_ values of every group in vitro test.

Concentration (µg•mL^−1^)	sGPS_1_	sGPS_2_	sGPS_3_	sGPS_4_	sGPS_5_
3.125	0.268±0.003^a^	0.199±0.004^d^	0.242±0.005^d^	0.270±0.006^b^	0.276±0.004^c^
1.563	0.244±0.004^b^	0.213±0.004^c^	0.282±0.006^c^	0.291±0.004^a^	0.301±0.005^b^
0.781	0.232±0.004^c^	0.242±0.004^b^	0.312±0.006^b^	0.277±0.005^ab^	0.326±0.004^a^
0.391	0.210±0.003^d^	0.270±0.005^a^	0.336±0.005^a^	0.272±0.007^b^	0.305±0.003^b^
0.195	0.195±0.005^e^	0.265±0.005^a^	0.295±0.004^c^	0.250±0.004^c^	0.275±0.005^c^
CC	0.192±0.001^e^	0.177±0.001^e^	0.204±0.004^e^	0.210±0.005^d^	0.200±0.003^d^

Concentration (µg•mL^−1^)	sGPS_6_	sGPS_7_	sGPS_8_	sGPS_9_	GPS
3.125	0.281±0.005^c^	0.201±0.002^cd^	0.346±0.004^a^	0.245±0.005^b^	0.264±0.005^a^
1.563	0.308±0.004^b^	0.224±0.004^b^	0.326±0.002^b^	0.257±0.003^ab^	0.253±0.004^a^
0.781	0.335±0.003^a^	0.236±0.004^a^	0.305±0.005^c^	0.272±0.004^a^	0.236±0.004^b^
0.391	0.320±0.003^b^	0.225±0.003^b^	0.293±0.005^d^	0.261±0.009^a^	0.229±0.004^b^
0.195	0.289±0.005^c^	0.210±0.003^c^	0.275±0.003^e^	0.265±0.007^a^	0.200±0.005^c^
CC	0.202±0.005^d^	0.195±0.004^d^	0.221±0.003^f^	0.210±0.002^c^	0.181±0.002^d^

Column data without the same superscripts (a–f) differ significantly (*P<*0.05). Values are mean ± standard error of the mean (SEM), *n* = 4/group.

**Figure 1:**
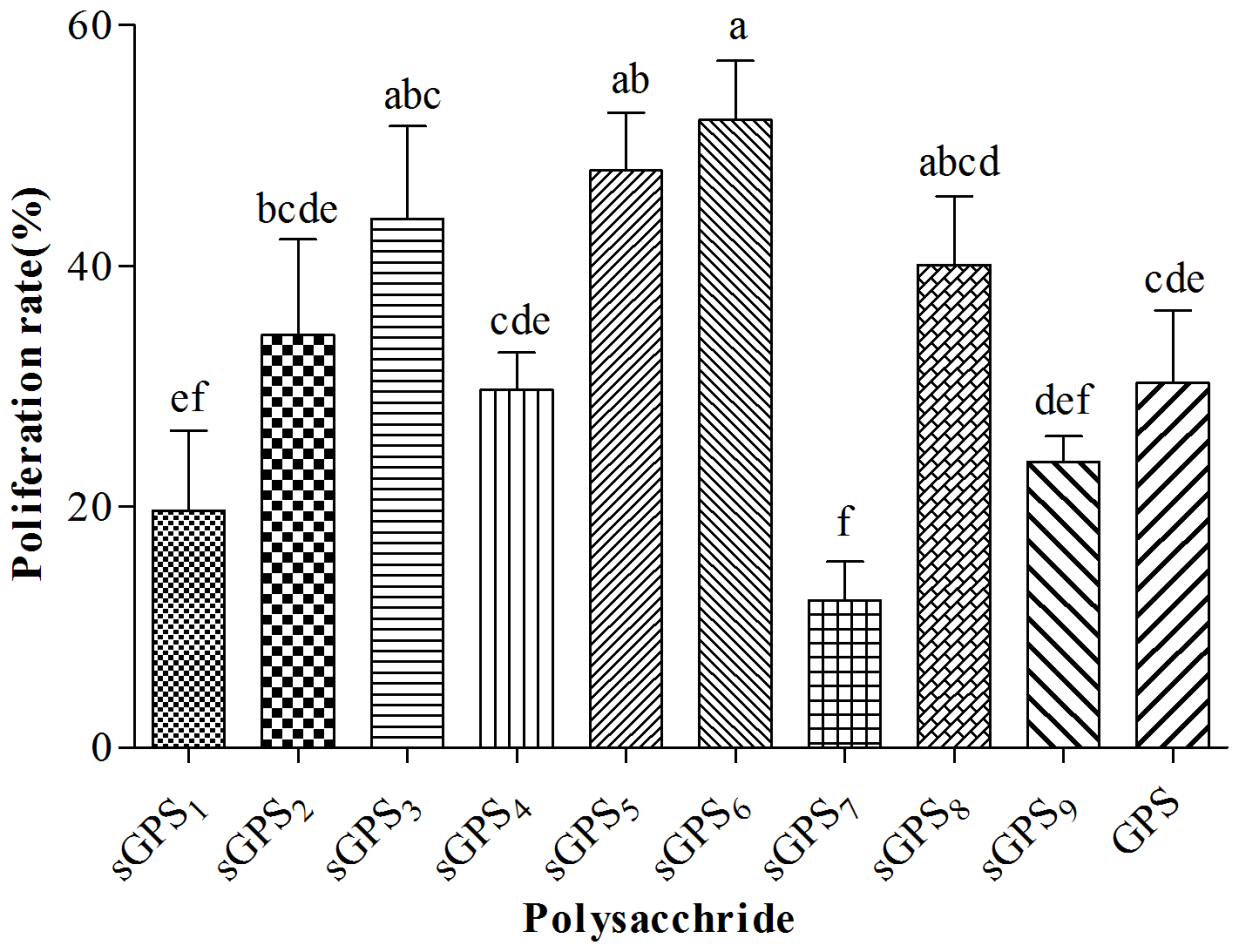
The lymphocyte proliferation rates of every group in vitro test. Bars without the same superscripts (a–f) differ significantly (*P*<0.05).

The lymphocytes proliferation rates of every group are illustrated in Fig. 1. The proliferation rate in sGPS_6_ group was the highest (52.16%), next was sGPS_5_ group (47.93%), these two groups were significantly higher than that in unmodified GPS group (*P<*0.05). In sGPS_3_ (43.90%), sGPS_8_ (40.11%) and sGPS_2_ (34.27%) groups were numerically higher than that in GPS group respectively (*P*>0.05).

### The changes of lymphocyte proliferation in vivo test

**Figure 2:**
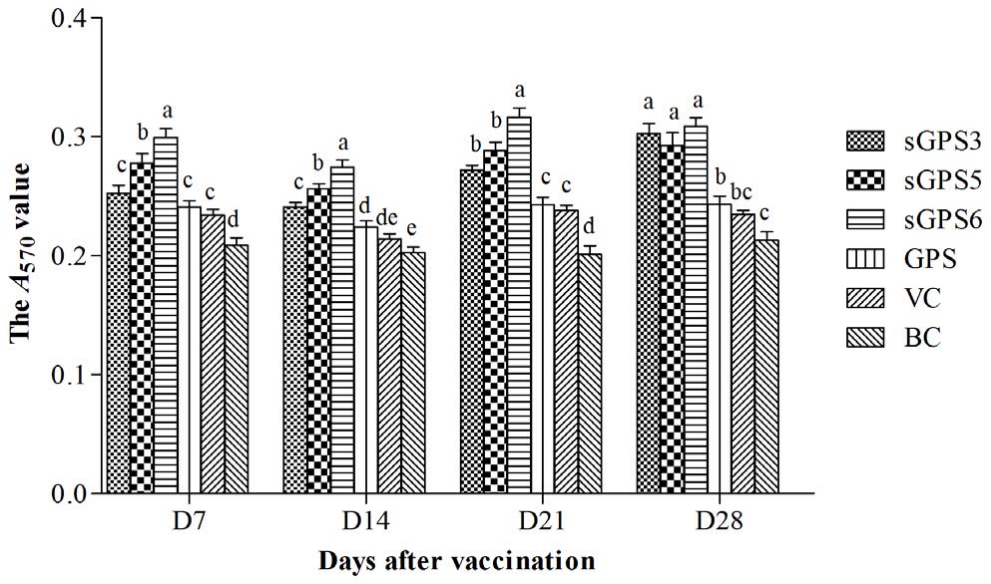
The cellular *A*_570_ values of every group in vivo test. Bars in same time point without the same superscripts (a–e) differ significantly (*P*<0.05).

The cellular *A*_570_ values of every group are illustrated in Fig. 2. At all time points after vaccination, the *A*_570_ values in all polysaccharide groups were significantly or numerically higher than those in VC and BC groups at same time points, in sGPS_6_ was the highest, and there were not significant differences between GPS group and VC group (*P*>0.05). The *A*_570_ values in sGPS_5_ and sGPS_6_ groups on D_7_ and in three sGPS groups on D_14_–D_28_ were significantly higher than those in VC and GPS group at same time points (*P*<0.05).

**Figure 3:**
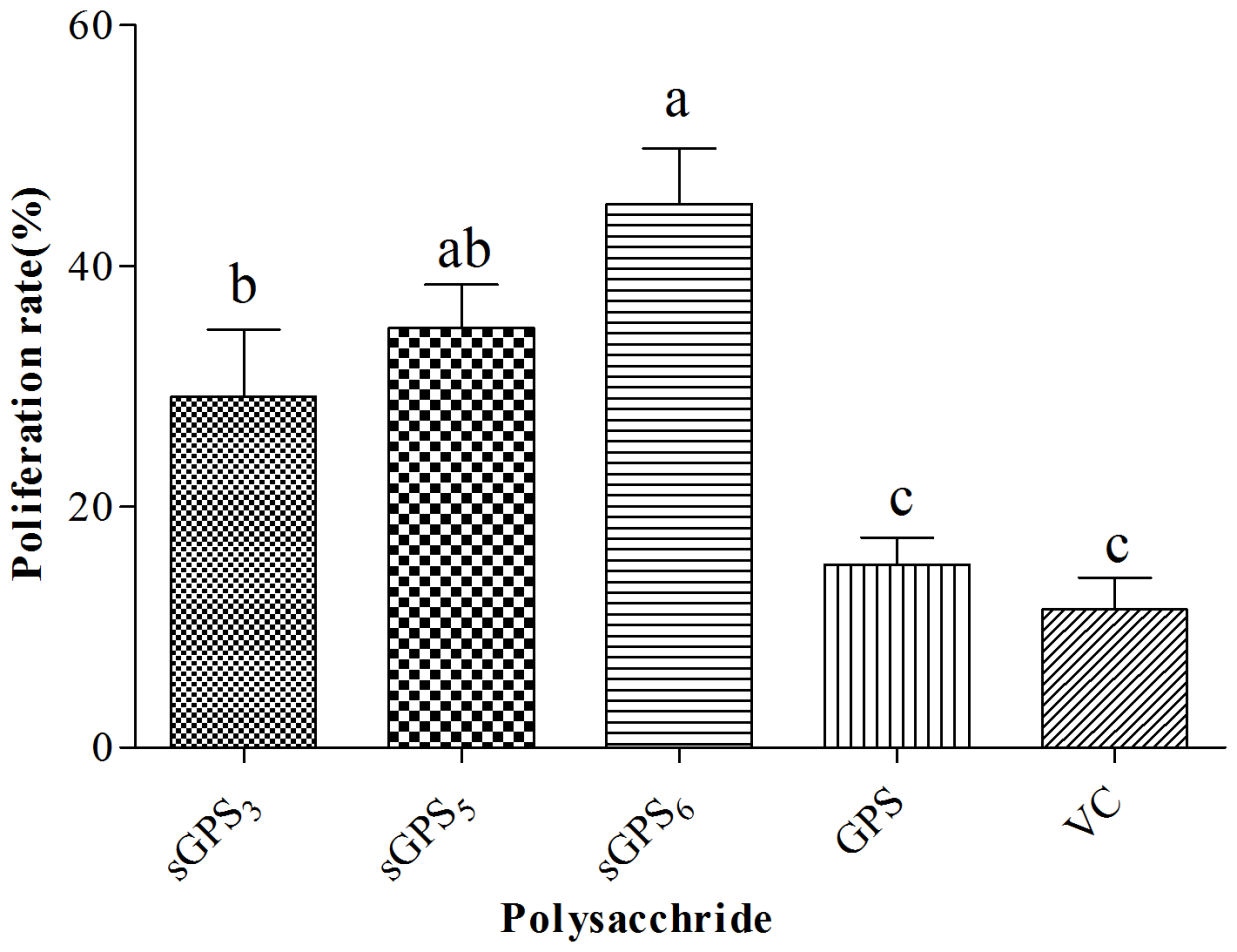
The average lymphocyte proliferation rates of every group in vivo test. Bars without the same superscripts (a–c) differ significantly (*P*<0.05).

The average lymphocyte proliferation rates of every group are illustrated in Fig. 3. The average lymphocyte proliferation rates in three sGPS groups were significantly higher than that of VC group and GPS group (*P*<0.05), in sGPS_6_ group was the highest (45.18%) and significantly higher than that of sGPS_3_ group (29.11%) (*P*<0.05). There was not significant difference between GPS (15.19%) group and VC (11.48%) group (*P*>0.05).

### The changes of serum antibody titer

**Figure 4:**
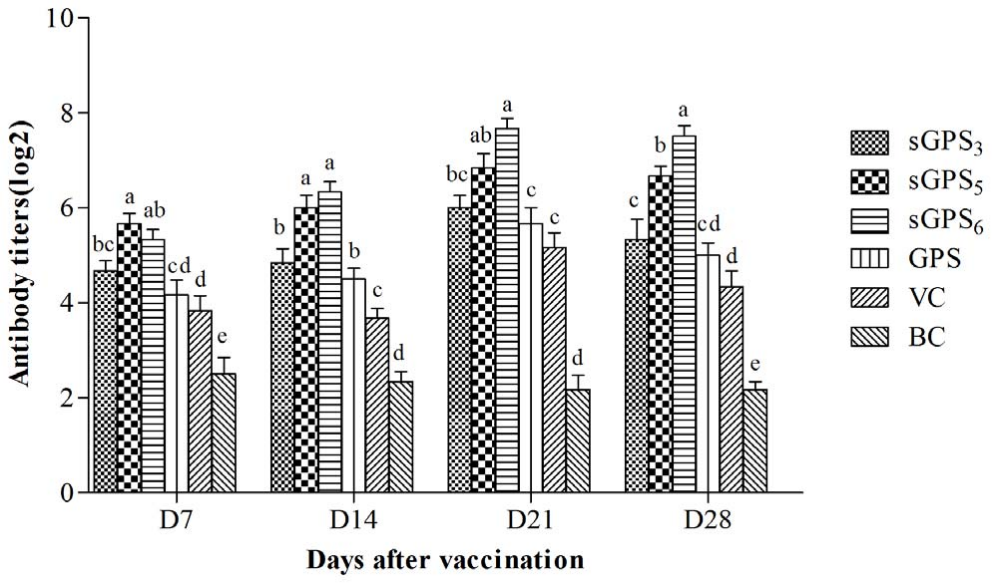
The antibody titers of every group in vivo test. Bars in same time point without the same superscripts (a–e) differ significantly (*P*<0.05).

The antibody titers of every group are illustrated in Fig. 4. At all time points after vaccination, the serum antibody titers in all polysaccharide groups were significantly or numerically higher than those in VC and BC groups, and in three sGPSs groups except sGPS_3_ on D_21_ were significantly higher than those in VC group (*P*<0.05). The antibody titers in sGPS_5_ group on D_7_ and sGPS_6_ group on D_14_–D_28_ were the highest, and in sGPS_5_ and sGPS_6_ groups at all time points were significantly higher than those in GPS group at same time points (*P*<0.05). The were not significant differences between sGPS_3_ and unmodified GPS group at all time points (*P*>0.05).

### The changes of serum IFN-γ contents

**Figure 5:**
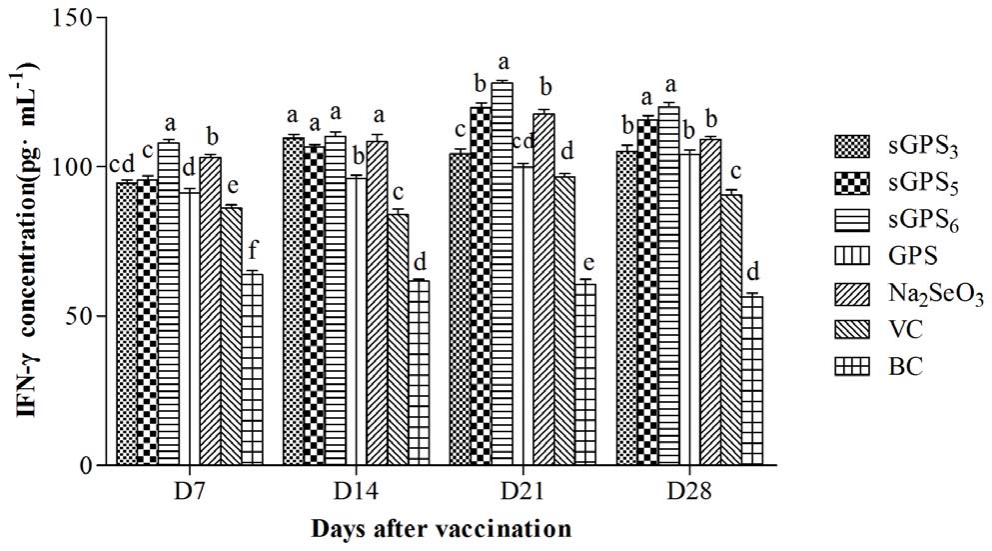
The serum IFN-γ contents of every group in vivo test. Bars in same time point without the same superscripts (a–e) differ significantly (*P*<0.05).

The serum IFN-γ contents of every group are illustrated in Fig. 5. At all time points after vaccination, the serum IFN-γ contents in all polysaccharide groups except GPS on D_21_ were significantly higher than those in VC and BC groups at same time points (*P*<0.05) and in sGPS_6_ group was the highest. The IFN-γ contents in sGPS_5_ and sGPS_6_ groups at all time points and in sGPS_3_ group on D_14_–D_21_ were significantly higher than those in GPS group at same time points (*P*<0.05).

### The changes of serum IL-2 contents

**Figure 6:**
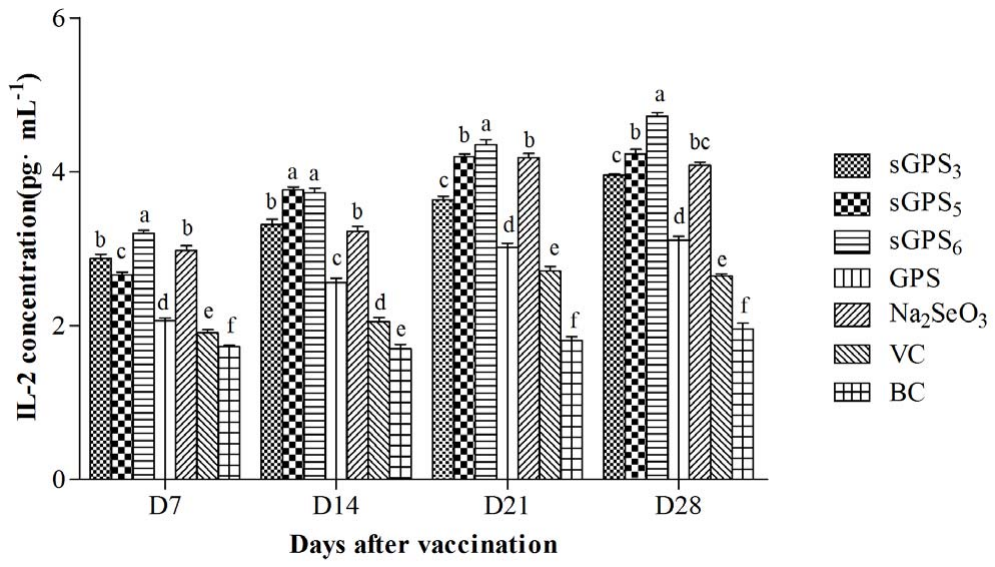
The serum IL-2 contents of every group in vivo test. Bars in same time point without the same superscripts (a–f) differ significantly (*P*<0.05).

The serum IL-2 contents of every group are illustrated in Fig. 6. At all time points after vaccination, the serum IL-2 contents in all polysaccharide groups were significantly higher than those in VC and BC groups (*P*<0.05), in sGPS_6_ group was the highest and in three sGPSs were significantly higher than those in GPS group at same time points (*P*<0.05).

## Discussion

As a safe and effective immune response regulator, selenium polysaccharide has the characteristics of low toxicity, high bioavailability, clear pharmacological action and so on [25]. It includes natural selenium polysaccharide and synthetic selenizing polysaccharide. Because of natural selenium polysaccharide with rare source, its application is restrained. In the past decades, people have been exploring the artificial methods to obtain selenium polysaccharide, such as selenic enrichment, selenic biotransformation, selenylation modification of polysaccharide, and so on [7], [26], [27]. Tang Jiajun et al synthesized a selenizing carrageenan polysaccharide with selenium powder and carrageenan [28]. Gong Xiaozhong prepared selenizing astragalus polysaccharide and selenizing dextran with high selenium content by selenylation reaction of the polysaccharides with a selenizing reagent with acyl chloride structure (SeOCl_2_) [29]. By comparison we found that nitric acid-sodium selenite method was an ideal method which was characterized by simple reaction process, feasible, less environmental pollution and convenient products recovery [14]. Therefore, this method was applied to modify garlic polysaccharide.

Immune response of organism includes cellular and humoral immunity. Cellular immunity is mediated mainly by T lymphocytes [30]. The lymphocyte proliferation is an important index to evaluate cellular immunity, and the lymphocyte proliferation rate can directly reflect the strength of cellular immunity [31]. The results in vitro test showed that the *A*_570_ values in sGPS_1_ at 3.125–0.391 µg·mL^−1^, sGPS_2_–sGPS_6_, sGPS_8_–sGPS_9_ and GPS at all concentrations and sGPS_7_ at 1.563–0.195 µg·mL^−1^ groups were significantly larger than those in corresponding cell control group, this indicated that they could significantly promote lymphocyte proliferation at these concentrations. The lymphocyte proliferation rate in sGPS_6_ group was the highest, next was sGPS_5_, these two groups were significantly and in sGPS_2_, sGPS_3_ and sGPS_8_ groups were numerically higher than that of unmodified GPS group, this indicated that selenylation modification could significantly enhance the cellular immune-enhancing activity of GPS in vitro and sGPS_6_ possessed the best efficacy.

The results in vivo test showed that at all time points after vaccination, the *A*_570_ values in all polysaccharide groups were significantly or numerically higher than those in VC groups at same time points, and the average lymphocyte proliferation rates in three sGPSs groups were significantly and in GPS group numerically higher than that of VC group, this indicated that these polysaccharides could promote lymphocyte proliferation. The *A*_570_ values in sGPS_5_ and sGPS_6_ groups on D_7_ and in three sGPS groups on D_14_–D_28_ were significantly higher than those in unmodified GPS group at same time points, in sGPS_6_ was the highest, there were not significant differences between GPS group and VC group, the average lymphocyte proliferation rates in three sGPS groups were significantly higher than that of unmodified GPS group (*P*<0.05) and in sGPS_6_ group was the highest, this indicated that selenylation modification could significantly enhance the cellular immune-enhancing activity of GPS in vivo, sGPS_6_ possessed the best efficacy. Liu Jia has confirmed that Se-EPS could promote macrophage-mediated immune reaction and boost immune response [32].

The humoral immune response mediated by B cells is a specific antigen-antibody reaction [33], and is one of the main factors to resist infectious diseases [34]. The antibody level is the marker reflected humoral immune function in bird species [35]. The results in vivo test showed that at all time points after vaccination, the serum antibody titers in all polysaccharide groups were higher than VC and BC groups, and in three sGPSs groups except sGPS_3_ on D_21_ were significantly higher than that in VC group, this indicated that these polysaccharides could significantly promote humoral immune response. The serum antibody titers in sGPS_5_ and sGPS_6_ groups at all time points were significantly higher than those in unmodified GPS group at same time points, and in sGPS_6_ group on D_14_–D_21_ was the highest, these results indicated that selenylation modification could significantly enhance the humoral immune-enhancing activity of GPS, sGPS_6_ possessed the best efficacy. Our previous research results also confirmed that selenylation modification can significantly enhance the immune-enhancing activity of CAP [36].

IFN-γ and IL-2 are secreted by Type 1 helper T cells (Th1 cell) mediating cellular immune response [37], and are the main cytokines that improve cell immunity. IL-2 can induce proliferation of B and NK cells, IFN-γ can activate macrophages and NK cells and promote the differentiation from TH0 cells into TH1 cells [38]–[40]. The results of this research showed that the serum IFN-γ contents in all polysaccharide groups except GPS on D_21_ were significantly higher than those in VC group at same time points, and the serum IL-2 contents in all polysaccharide groups at all time points were significantly higher than those in VC group at same time points, these results indicated that they could significantly promote secretion of these two cytokines, thereby enhancing immunity. The IFN-γ contents in sGPS_5_ and sGPS_6_ groups at all time points and in sGPS_3_ group on D_14_–D_21_ were significantly higher than those in unmodified GPS group, the serum IL-2 contents in three sGPSs were significantly higher than that in unmodified GPS group, and the serum IFN-γ and IL-2 contents in sGPS_6_ were the highest, these results indicated that selenylation modification could significantly promote secretion of cytokines, sGPS_6_ possessed the best efficacy. Other study confirmed that Se-ECZ-EPS could significantly enhance the contents of cytokine compared with Na_2_SeO_3_.

Selenizing polysaccharide is synthesized with selenium and polysaccharide therefore possesses dual pharmacological activities of selenium and polysaccharide but it is not the simple addition of two kinds of activities, its activity is higher or newer as compared with selenium and polysaccharide [26], [41]–[42]. This study found that the immune-enhancing activity of sGPSs has certain correlation with its selenium content and carbohydrate content. Among the nine sGPSs, sGPS_3_, sGPS_5_ and sGPS_6_ presented stronger immune-enhancing activity in vitro, their selenium or carbohydrate contents were located in the first two places. sGPS_6_ presented the strongest immune-enhancing activity in vivo, its selenium and carbohydrate contents were arranged in second place, while sGPS_3_ had the highest selenium content and the lowest carbohydrate content, and sGPS_5_ had the highest carbohydrate content, the lowest selenium content, their immune-enhancing activities were lower than that of sGPS_6_. From this it could be seen that selenizing polysaccharide with stronger immune-enhancing activity required the combination of higher selenium content with higher carbohydrate content.

In conclusion, selenylation modification can significantly enhance the immune-enhancing activity of GPS, sGPS_6_ presented best efficacy and would be as a candidate drug of new-type immunoenhancer, the optimal modification conditions were 400 mg of sodium selenite for 500 mg of GPS, the reaction temperature of 70°C and the reaction time of 6 h.
